# Calprotectin Levels and Neutrophil Count Are Prognostic Markers of Mortality in COVID-19 Patients

**DOI:** 10.3390/diagnostics12102554

**Published:** 2022-10-20

**Authors:** Giovanna Cardiero, Daniela Palma, Martina Vano, Claudia Anastasio, Biagio Pinchera, Martina Ferrandino, Carlo Gianfico, Luca Gentile, Marcella Savoia, Ivan Gentile, Maria Donata Di Taranto, Giuliana Fortunato

**Affiliations:** 1 Dipartimento di Medicina Molecolare e Biotecnologie Mediche, Università degli Studi di Napoli Federico II, 80131 Naples, Italy; 2 CEINGE-Biotecnologie Avanzate Franco Salvatore, 80145 Naples, Italy; 3 Dipartimento di Medicina Clinica e Chirurgia, Sezione di Malattie Infettive, Università degli Studi di Napoli Federico II, 80131 Naples, Italy

**Keywords:** COVID-19, calprotectin, S100A8/S100A9, neutrophil, inflammation, biomarker, survival, prognosis

## Abstract

Inflammation plays a crucial role in worsening coronavirus disease (COVID-19). Calprotectin is a pro-inflammatory molecule produced by monocytes and neutrophilic granulocytes. The aim of the study was to evaluate both the prognostic role of circulating calprotectin levels and neutrophil count toward fatal outcome in COVID-19 patients. We retrospectively collected and analyzed data on 195 COVID-19 adult patients, 156 hospitalized in the infectious disease unit and 39 in the intensive care unit (ICU). Calprotectin levels and neutrophil counts measured at the first hospitalization day were higher in the patients with a fatal outcome than in surviving ones. The association of high calprotectin levels and neutrophil count to patient death remain significant by logistic regression, independent of patient age. ROC curves analysis for calprotectin levels and neutrophil count revealed a good discriminatory power toward survival (area under the curve of 0.759 and 0.843, respectively) and identified the best cut-off (1.66 mg/L and 16.39 × 10^3^/µL, respectively). Kaplan–Meier analysis confirmed the prognostic role of high calprotectin levels and neutrophil count in death prediction. In conclusion, this study highlights that calprotectin levels together with neutrophil count should be considered as biomarkers of mortality in COVID-19 patients.

## 1. Introduction

The hallmark of the severe form of coronavirus disease (COVID-19) is an uncontrolled inflammatory response of the host due to the so-called “cytokine storm” that generates a systemic inflammatory status [1,2]. The cytokine storm is considered one of the main causes of acute respiratory distress syndrome (ARDS) and multi-organ failure that characterize the severe form of the infection and are responsible for most deaths.

Several biomarkers have been investigated for their potential to predict a fatal outcome in COVID-19 patients [3,4,5]. As inflammation plays a crucial role in disease worsening, the early detection of the inflammatory status, as well as pharmacological treatment aimed at suppressing inflammatory response, are fundamental to prevent the worsening of COVID-19 infected patients, and to save patients’ lives [1]. Consequently, the search for possible independent predictors allowing for early risk stratification and the management of COVID-19 patients has been focused on inflammatory biomarkers. Several studies have shown that classic inflammatory markers, such as C reactive protein (CRP), procalcitonin, D-dimer, LDH and ferritin, are significantly associated with a poor prognosis in COVID-19 patients [6,7,8].

Calprotectin is a calcium-binding heterodimer (S100A8/S100A9) of 24 kDa, which plays an important role in the inflammatory response [9] and whose levels have been shown to be significantly increased in patients with various inflammatory and autoimmune conditions [10,11].

Calprotectin is produced by monocytes and neutrophilic granulocytes, of which it constitutes about half of their total cytosolic protein content [9]. In the presence of inflammatory processes, calprotectin is released by neutrophils and triggers the inflammatory response.

Unlike other routinely used inflammatory biomarkers, such as CRP and procalcitonin, calprotectin is released by neutrophils into the bloodstream without requiring de novo protein biosynthesis [12].

In the last year, many studies addressed the role of calprotectin as predictive biomarker of COVID-19 severity [13,14,15], including a meta-analysis [16]. However, the prognostic role of calprotectin as a biomarker in COVID-19 patients remains unclear [17]. The aim of the study was to evaluate both the prognostic role of circulating calprotectin and neutrophil count toward fatal outcome in COVID-19 patients.

## 2. Materials and Methods

### 2.1. Patients, Samples and Biochemical Analyses

We retrospectively collected and analyzed data from 195 COVID-19 adult patients, 156 hospitalized in the infectious disease unit and 39 in the intensive care unit (ICU) of the University Hospital Federico II, from 10 March 2020 to 30 April 2020, and from 8 December 2020 to 30 April 2021. As to the 39 ICU patients, we only considered patients immediately admitted to ICU, without a previous hospitalization.

The study was performed according to the current version of the Helsinki Declaration and was approved by the Ethical Committee of the University Hospital Federico II (Number 156/20, 22 April 2020). Informed consent for the study was obtained from each patient.

A confirmed case of COVID-19 was defined by a positive result on molecular analysis with a reverse transcriptase polymerase chain reaction (RT-PCR) on nasopharyngeal swab. Exclusion criteria included the inability to understand or sign informed consent, age <18 years, absence of positivity for SARS-CoV-2 RNA test and positivity for serological tests only.

EDTA-plasma and serum samples were collected from each patient on the first hospitalization day. The anamnestic data of patients (e.g., presence of comorbidities) as well as data on the COVID-19 clinical course before hospitalization were acquired. Patients were followed until discharge/death.

Plasma calprotectin and serum CRP levels were measured by a particle enhanced turbidimetric immunoassay (PETIA) (GentianAS, Norway) and a latex immunoassay with a high-sensitivity method (Sentinel Diagnostics, Milano, Italy), respectively, according to the manufacturer’s recommendations. The ACHITECT i2000R System (Abbott Laboratories, Wiesbaden, Germany) was used for measurements. Neutrophil count was evaluated by standard blood count on the ADVIA 2120 Hematology System (Siemens Healthcare).

### 2.2. Statistical Analysis

The normality of variable distribution was evaluated using the Kolmogorov-Smirnov test. Since all continuous variables did not show a normal distribution, they were reported as median value and interquartile range and compared by the Mann-Whitney test. Spearman correlation was used to test the relationship between the different variables. Different logistic regression models were created to analyze the role of the different biochemical variables, which resulted to be correlated; all models included sex and age. Additional logistic regression models were evaluated including sex, age, the presence of decreased lung function, diabetes, heart disease, hypertension, and the number of hospitalization days. Model 1 included calprotectin circulating levels, model 2 included CRP levels, and model 3 included neutrophil count. Odd Ratios (OR) and their 95% Confidence Interval (95% CI) were reported when significant. A *p* value <0.05 was considered significant. Statistical analyses were carried out using PASW version 27 (SPSS Inc., Chicago, IL, USA). The MedCalc version 20.027 was used for ROC curve analysis. The statistical significance of the area under the ROC curves (AUC) was calculated against the null hypothesis of an AUC = 0.5 (area under the bisector). The best cut-off was identified as the farthest point from the bisector. Comparison between AUC was performed by the DeLong test.

## 3. Results

### 3.1. Biomarker Association to Fatal Outcome

Among the 195 patients (101 women and 94 men) studied, 10 patients experienced a fatal outcome in-hospital, all from the ICU. Circulating calprotectin was measured on the first hospitalization day in all patients, together with other inflammatory markers such as CRP and neutrophil count. Table 1 shows biochemical and clinical features of the studied population divided based on the outcome.

Calprotectin levels and neutrophil counts were higher in patients with a fatal outcome than in surviving ones, whereas CRP levels were not statistically different. We verified that the levels of these three markers did not differ between patients from either ICU or the infectious disease unit. A logistic regression analysis was performed in order to evaluate these associations independent of age and sex. Since calprotectin levels correlated with the levels of the other inflammation markers CRP (Spearman coefficient 0.354, *p* < 0.001) and neutrophil count (Spearman coefficient 0.350, *p* < 0.001), we created different logistic regression models to avoid collinearity issues (Table 2). Both calprotectin levels and neutrophil counts were associated with survival independent of age with an OR (95% CI) of 1.874 (1.312–2.677) and 1.364 (1.163–1.600), respectively (Table 2). Considering only the 39 patients from the ICU, similar results were observed, being the association to death significant for calprotectin levels with an OR (95% CI) of 2.422 (1.199–4.891)—*p* = 0.014—and for neutrophil count with an OR (95% CI) of 1.266 (1.063–1.507)—*p* = 0.008; no association was found for CRP levels-OR (95% CI) of 1.002 (0.989–1.016) with *p* = 0.757.

Despite not being recommended for a low event number, a logistic regression also including the presence of reduced lung function, diabetes, heart disease and hypertension was performed. Both calprotectin levels and neutrophil counts were associated with survival independently of the other parameters with an OR (95% CI) of 1.843 (1.292–2.630) and 1.466 (1.184–1.816), respectively.

### 3.2. Identification of Cut-Off and Survival Analysis

**Figure 1:**
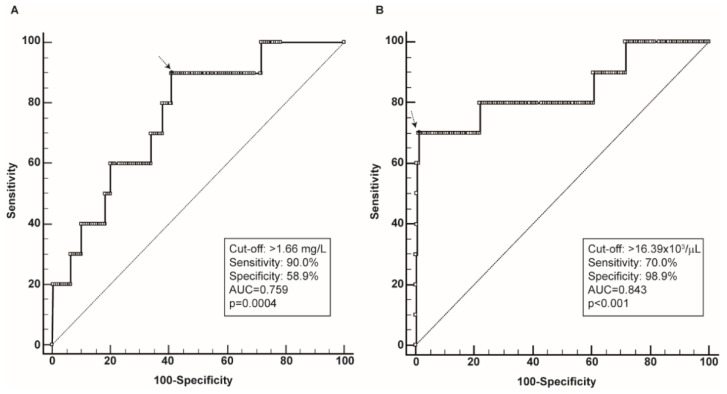
ROC curves of calprotectin levels and neutrophil count in relation to survival. The ROC curve is indicated by a bold line and full circles represent the different criterion points. The open circle indicates the best cut-off point. The dotted line indicates the bisector. AUC = area under the curve. (**A**). ROC curve of calprotectin levels. (**B**). ROC curve of neutrophil count.

The ROC curve analysis of calprotectin levels and neutrophil counts evaluated at the beginning of hospitalization revealed a good discriminatory power toward survival with an AUC of 0.759 (*p* = 0.0004) and 0.843 (*p* < 0.0001), respectively; the difference of AUCs was not statistically significant (Figure 1). The best cut-off value for calprotectin was 1.66 mg/L with a sensitivity of 90% and specificity of 58.9%, whereas the best cut-off for neutrophil count was 16.39 × 10^3^/µL with a sensitivity of 70% and a specificity of 98.9%.

**Figure 2:**
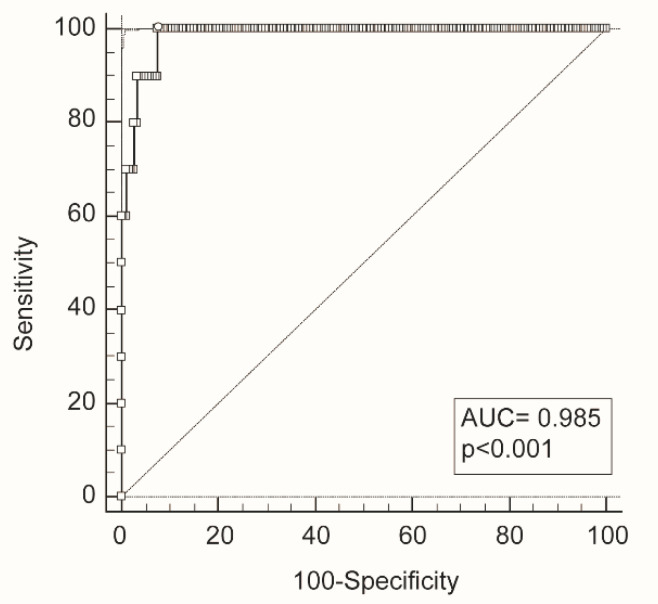
ROC curve of age, calprotectin levels and neutrophil count combined together. The weight of each parameter was obtained by logistic regression. The ROC curve is indicated by a bold line and full circles represent the different criterion points. The open circle indicates the best cut-off point. The dotted line indicates the bisector. AUC = area under the curve.

To verify the discrimination power of age, calprotectin levels and neutrophil counts combined together we constructed a ROC curve considering the weight of each parameter as determined by logistic regression (Figure 2). The very high AUC indicates that survival prediction can be improved considering together these three parameters.

**Figure 3:**
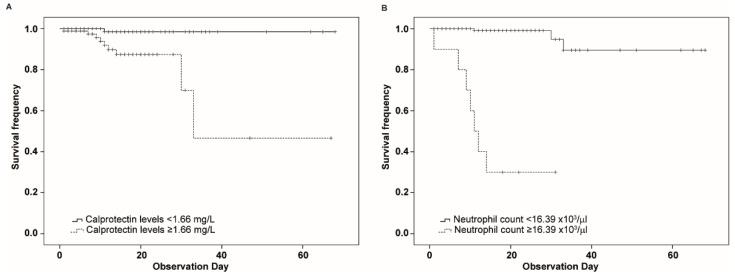
Kaplan–Meier survival curves of patients in relation to calprotectin levels or neutrophil count. (**A**) Patients were divided based on calprotectin levels higher or lower than 1.66 mg/L at the first measurement. (**B**) Patients were divided based on neutrophil count higher or lower than 16.39 × 10^3^/µL at the first measurement. Each vertical drop of the curve represents a single event. Plus signs indicate censored cases.

To test the prognostic value of calprotectin levels and neutrophil count toward exitus, a Kaplan–Meier analysis was performed grouping the patients according to the identified cut-offs (1.66 mg/L for calprotectin and 16.39 × 10^3^/µL for neutrophil count). For calprotectin, the log-rank test revealed a statistical difference in terms of survival between the two groups (*p* < 0.0005), calprotectin levels higher than 1.66 mg/L being a predictor of death (Figure 3). As to the neutrophil count, the survival was lower in the group of patients with a neutrophil count higher than 16.39 × 10^3^/µL (*p* < 0.0001—Figure 3).

**Figure 4:**
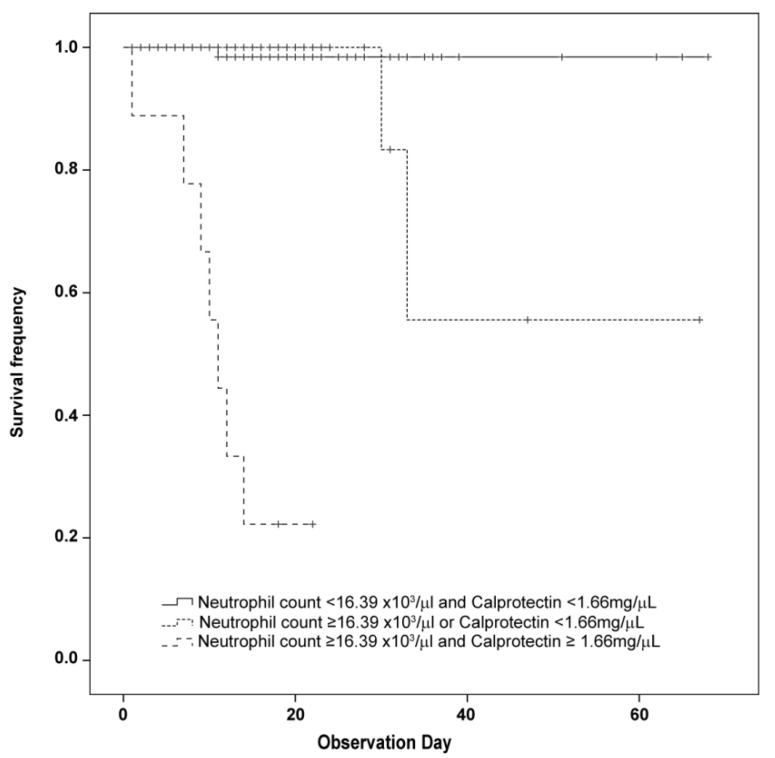
Kaplan–Meier survival curves of patients in relation to both calprotectin levels and neutrophil count. Patients were divided based on levels over the cut-off for both calprotectin and neutrophil count, levels over the cut-off for only one of the parameters or levels below the cut-off for both parameters. Calprotectin cut-off at the first measurement: 1.66 mg/L. Neutrophil count cut-off at the first measurement: 16.39 × 10^3^/µL. Each vertical drop of the curve represents a single event. Plus signs indicate censored cases.

A Kaplan–Meier analysis was performed considering the three groups defined by levels over the cut-off for both calprotectin and neutrophil count, levels over the cut-off for only one of the parameters or levels below the cut-off for both parameters (Figure 4). Data showed that levels of calprotectin and neutrophil count exceeding the identified thresholds can synergically contribute to the identification of non-surviving patients (*p* < 0.000001).

## 4. Discussion

Inflammation is a biological process triggering the clinical worsening of SARS-CoV-2 infection. Several studies have been conducted to validate the use of inflammatory biomarkers to predict the severity of COVID-19 clinical course [18,19]. Since neutrophil activation is the phenomenon triggering inflammation, neutrophil-related biomarkers, including calprotectin, were given great consideration for the management of COVID-19 patients.

Our retrospective study has highlighted that calprotectin and neutrophil count may be useful markers of fatal outcome in COVID-19 patients, even more useful than CRP. Other studies investigated the association of calprotectin levels with patient mortality [20,21,22,23]. Two of these revealed that non-surviving COVID-19 patients showed increased calprotectin levels than surviving ones [20,21]. Nevejan and colleagues [22] did not find any association with mortality but only with COVID-19 severity. A further study [23] did not find this kind of association probably due to the low number of studied patients (*n* = 19). Several other studies also revealed the predictive role of high calprotectin levels toward COVID-19 severity [24,25,26]. In addition, several other studies were focused on the predictive role of calprotectin toward COVID-19 severity revealing.

Our study is based on a population of 195 patients from both the infectious disease unit and the ICU, followed for the whole hospitalization time. Both calprotectin levels and neutrophil counts were higher in non-surviving patients than surviving ones. The multivariate logistic regression showed that these associations remain statistically significant independent of age, sex and the presence of co-morbidities such as diabetes, heart disease, hypertension and decreased lung function. In particular, a higher OR was observed for the association of calprotectin levels with mortality (OR = 1.843 (95% CI: 1.292–2.630)) and with respect to neutrophil count (OR = 1.466 (95% CI: 1.184–1.816)).

Furthermore, an analysis of ROC curves established that a threshold of 1.66 mg/L allows the detection of patients undergoing a fatal outcome with 90% of sensitivity, although this low threshold is associated with a low specificity (58.9%). Similar sensitivity and specificity related to calprotectin levels were also observed by Ducastel et al. [20], although the thresholds identified in different studies cannot be compared as the studies were based on different methods and biological matrices. The AUC of neutrophil count revealed an even better discriminatory power (AUC = 0.843), although the identified threshold of 16.39 × 10^3^/µL showed a low sensitivity (70%) and a very high specificity (98.9%).

The higher sensitivity of calprotectin with respect to neutrophil count could be explained by the fact that calprotectin is an indicator of neutrophil activation, a phenomenon more related to the inflammatory response than the number of neutrophils, including quiescent cells. Several studies investigated white blood cell alterations in COVID-19 patients and in particular neutrophil count [27,28]. To the best of our knowledge there is no other study comparing the performance of neutrophil count and calprotectin levels in the mortality prediction/for predicting mortality in COVID-19 patients, whereas the correlation between neutrophil count and calprotectin levels was already observed [26,29].

Kaplan-Meier analysis confirmed the usefulness of both calprotectin levels and neutrophil count in mortality prediction. In fact, patients with calprotectin levels higher than 1.66 mg/L as well as patients with a neutrophil count higher than 16.39 × 10^3^/µL showed a much poorer prognosis than patients with lower levels. Furthermore, considering both parameters at the same time, the identification of patients at high risk of death is improved. In fact, an additive effect was observed for both neutrophil count and calprotectin levels over the threshold in the identification of non-surviving patients.

Several recent reports about the usefulness of calprotectin level measurements have been published [13,14,15,16]. Reported differences in the association of high calprotectin levels to fatal outcome can be explained by the differences in the features of the studied population (such as age, co-morbidity, death rate, etc.). The limitations of the study are related to the small sample size, the retrospective study design, and the necessity to consider reported data generalizable to the specific group of patients. This report highlights the role of calprotectin levels in survival prediction on a population of relatively young patients with low systemic inflammation. Despite the number of analysed subjects not being high, this study can contribute to future meta-analysis focused on the calprotectin prognostic role [16,24].

However, the calprotectin threshold we identified should be considered valid under the analytical conditions used. Values observed in COVID-19 patients largely varied according to different studies using different methodologies. A recent paper [22] pointed out the value differences related to the biological matrix used for calprotectin measurement. In particular, calprotectin serum levels are higher than plasma levels because the platelet activation leads to neutrophil damage and “in tube” calprotectin release. Therefore, data reported in this study should be considered specific to the used measurement method and sample matrix, i.e., EDTA-plasma. An improvement of harmonization in the measurement of circulating calprotectin could highlight the real value of this marker in COVID-19 as well as in other inflammatory diseases, such as autoimmune diseases [30,31] and atherosclerosis-based cardiovascular diseases [32,33,34]. Another point that should be addressed to improve the adoption of calprotectin in routine analyses is related to the timing of calprotectin increase before the worsening of diseases. This aspect was pointed out by recent papers focused on COVID-19 [13,14].

In conclusion, this study highlights that calprotectin levels together with neutrophil count should be considered as biomarkers of mortality in COVID-19 patients.

## Figures and Tables

**Table 1:** Demographic and biochemical inflammatory parameters of studied population.

	Total (*n* = 195)	Surviving (*n* = 185)	Non-Surviving (*n* = 10)	Significance
Age, years	55 (34–67)	51 (33–66)	77 (62–85)	*p* < 0.001
Sex, number of males (%)	94 (48.2%)	86 (46.5%)	8 (80.0%)	ns
Serum Calprotectin (mg/L)	1.32 (0.58–3.91)	1.28 (0.54–3.60)	4.26 (2.01–9.84)	*p* = 0.006
C-reactive protein (mg/L)	17.8 (3.73–54.64)	16.80 (3.60–47.92)	62.16 (8.45–78.62)	ns (*p* = 0.127)
Neutrophil count (×10^3^/µL)	6.38 (4.80–9.07)	6.26 (4.73–8.49)	20.26 (8.29–30.26)	*p* = 0.0003
Decreased lung function, *n*, (%)	170 (87.2%)	164 (88.6%)	6 (60.0%)	*p* = 0.026
Diabetes *n*, (%)	155 (79.5%)	149 (80.5%)	6 (60.0%)	ns
Heart disease *n*, (%)	187 (95.9%)	179 (96.8%)	8 (80.0%)	ns
Hypertension *n*, (%)	145 (74.4%)	139 (75.1%)	6 (60.0%)	ns
Hospitalization days	13 (7–21)	13 (7–21)	11 (8–18)	ns

Continuous data are reported as median value with interquartile range in parentheses. Frequencies are reported as absolute number and percentage in parentheses. ns: not significant.

**Table 2:** Results of multivariate logistic analyses.

Variables	Model 1 OR (95% CI) *significance*	Model 2 OR (95% CI) *significance*	Model 3 OR (95% CI) *significance*
Sex	ns	ns	ns
Age	1.186 (1.059–1.329) *p = 0.003*	1.085 (1.026–1.148) *p = 0.004*	1.111 (1.018–1.213) *p = 0.018*
Calprotectin levels	1.874 (1.312–2.677) *p = 0.001*	-	-
C-reactive protein	-	1.002 (0.992–1.013) ns (*p* = 0.678)	-
Neutrophil count	-	-	1.364 (1.163–1.600) *p < 0.001*

OR: odds ratio; CI: confidence interval; ns: not significant.

## Data Availability

The data presented in this study are available on request from the corresponding author.
